# Two new species of *Pleosporales* from northern Thailand: *Anastomitrabeculia chiangraiensis* sp. nov. (*Anastomitrabeculiaceae*) and *Roussoella chiangraiensis* sp. nov. (*Roussoellaceae*)

**DOI:** 10.3897/mycokeys.138.189855

**Published:** 2026-08-07

**Authors:** Yu Xiao, Zaw Lin Tun, Yong Wang, Deecksha Gomdola, Long-Feng Yu

**Affiliations:** 1 College of Biotechnology and Engineering, West Yunnan University, Lincang, Yunnan 677000, China Center of Excellence in Fungal Research, Mae Fah Luang University Chiang Rai Thailand; 2 Department of Plant Pathology, College of Agriculture, Guizhou University, Guiyang, Guizhou 550025, China School of Science, Mae Fah Luang University Chiang Rai Thailand; 3 School of Science, Mae Fah Luang University, Chiang Rai 57100, Thailand Department of Plant Pathology, College of Agriculture, Guizhou University Guiyang China; 4 Center of Excellence in Fungal Research, Mae Fah Luang University, Chiang Rai 57100, Thailand College of Biotechnology and Engineering, West Yunnan University Lincang China

**Keywords:** *
Ascomycota
*, morpho-molecular, species delineation, systematics, two new species

## Abstract

*Pleosporales* is a highly diverse order in *Dothideomycetes*, with numerous taxa occupying saprobic, pathogenic and endophytic niches worldwide. During a survey of microfungi in northern Thailand, two sexual morph species were collected and examined, based on morphology and multigene phylogenetic analyses (28S, ITS, 18S, *tef1-α* and *rpb2*). Based on this approach, two new species were introduced. *Anastomitrabeculia chiangraiensis***sp. nov**., isolated from a dead stem of an unidentified woody host, forms a distinct lineage within *Anastomitrabeculiaceae* and can be distinguished from its sister taxon, *A. didymospora*, by the absence of longitudinal striations in the ascospores and high interspecies sequence divergence in the ITS, 28S and *rpb2* regions. *Roussoella chiangraiensis***sp. nov**., collected from dead bamboo stems, is well-resolved within *Roussoellaceae* and differs from the closely-related *R. aseptata* with robust phylogenetic support (100% ML, 1.00 PP) and significant interspecies genetic distance in ITS. Morphological differentiation from *R. aseptata* was restricted due to the absence of a known sexual morph for that species. Our findings highlight the high, yet underexplored diversity of saprobic *Pleosporales* in Southeast Asia and underscores the importance of integrative morphological and phylogenetic approaches for accurate species recognition.

## Introduction

*Pleosporales* is the largest order within *Dothideomycetes* and encompasses a wide array of ecologically diverse ascomycetes (Zhang et al. 2012; Hongsanan et al. 2020). Pleosporalean taxa occur in a broad range of habitats and exhibit saprobic, pathogenic, endophytic and epiphytic lifestyles, with a cosmopolitan distribution (Zhang et al. 2012; Hyde et al. 2013; Hongsanan et al. 2020). As saprobes, these fungi play critical roles in nutrient cycling and organic matter decomposition, particularly in terrestrial ecosystems (O’Hanlon 2017). As plant pathogens, several species are of economic importance, causing leaf spots, blights and stem diseases in crops and natural forest ecosystems (Doehlemann et al. 2017). Endophytic and epiphytic taxa further contribute to the ecological versatility of the order, colonising living plant tissues without causing apparent harm, while some groups are associated with freshwater or marine substrates (Hyde et al. 2019). This range of ecological strategies is matched by substantial morphological and genetic diversity, making *Pleosporales* one of the most taxonomically complex and species-rich groups in *Dothideomycetes* (Hongsanan et al. 2020).

Taxonomic studies and species discovery in *Pleosporales* continue to advance rapidly, driven by increasing use of integrative approaches that combine detailed morphological examination with multigene phylogenetic analyses. Recent studies have revealed numerous novel fungal taxa in families such as *Anastomitrabeculiaceae*, *Corynesporascaceae* and *Roussoellaceae*, particularly from tropical and subtropical regions, harbouring a high fungal diversity (Bhunjun et al. 2021; Ren et al. 2024; Yu et al. 2024; Sun et al. 2025). Northern Thailand, with its rich plant biodiversity, is recognised as a hotspot for microfungal diversity (Hyde et al. 2018). The region’s extensive bamboo forests and mixed deciduous vegetation provide suitable substrates for saprobic taxa (Dai et al. 2017; Hyde et al. 2018), including many species that produce immersed or semi-immersed ascomata and are, therefore, easily overlooked in field surveys.

The aim of this study is to describe and illustrate two novel Pleosporalean species, one belonging to *Anastomitrabeculia* and the other to *Roussoella*, based on morphological examination and multigene phylogenetic analyses. This study contributes to ongoing efforts to document the taxonomic richness of Pleosporales and underscores the importance of continued mycological surveys in biodiversity hotspots such as northern Thailand.

## Material and methods

### Sample collection and examination

Dead stems of unidentified woody hosts and bamboo were collected from Mae Fah Luang University premises, Chiang Rai Province, Thailand. Samples were collected in plastic bags and taken to the laboratory for further examination (Senanayake et al. 2020). Fungal macro- and micro-morphological characters were observed using a Motic SMZ-168 stereomicroscope. Single-spore isolation followed Senanayake et al. (2020), although no spores germinated on artificial media. Microscopic images were captured with a Canon 750D camera mounted on a Nikon ECLIPSE E600 compound microscope under bright-field illumination. Photoplates were assembled in Adobe Photoshop CS6 (v.2023) and ungal structures were measured in Tarosoft Image Framework (v.0.97).

Holotype materials are deposited in the Mae Fah Luang University Herbarium (MFLU). Faces of Fungi and Index Fungorum numbers were assigned to the newly introduced species (Jayasiri et al. 2015; Index Fungorum 2026). Descriptions and illustrations of the taxa are also incorporated into the GMS Microfungi Database (Chaiwan et al. 2021). New taxa were established following the morphological and phylogenetic species concepts outlined in Chethana et al. (2021). Taxonomic placement follows the most recent classification by Hyde et al. (2024), Index Fungorum (2026) (https://www.indexfungorum.org/Names/Names.asp) and Species Fungorum (2026) (https://www.speciesfungorum.org/Names/Names.asp).

### DNA extraction, PCR amplification and sequencing

Total genomic DNA was extracted directly from ascomata. Approximately 15–20 ascomata were picked from the substrates under a Motic SMZ-168 stereomicroscope using a sterile needle and transferred into 1.5 ml microcentrifuge tubes. DNA extraction was carried out using the Fo­rensic DNA Kit (D3396-01, OMEGA Bio-Tek, Inc., Winooski, VT, USA), following the manufacturer’s protocol. The amplified gene regions in this study are listed in Table 1.

**Table 1.** Primers used in this study.

**Gene regions**	**Primer pairs**	**References**
ITS; internal transcribed spacer (ITS, nuclear rDNA consisting of ITS1-5.8S-ITS2)	ITS4/ITS5	White et al. (1990)
28S; large subunit (28S, D1–D2 domains of nuclear 28S rDNA)	LR0R/LR5	White et al. (1990)
18S; small subunit (18S, partial or complete nuclear 18S rDNA)	NS1/NS4	White et al. (1990)
*rpb2*; RNA polymerase 2	5F2/7CR	Liu et al. (1999)

The polymerase chain reaction (PCR) mixture was prepared in a final volume of 25 μl, containing 12.5 μl of GoTaq Green Master Mix (Promega), 1 μl of each forward and reverse primers (10 µM), 2 μl of genomic DNA and 8.5 μl of distilled water. Thermal cycling conditions consisted of an initial denaturation at 95 °C for 3 min; followed by 40 cycles of 95 °C for 45 s, annealing at 55 °C for ITS, 52 °C for 28S and 18S and 58 °C for *rpb2* (each for 50 s, except *rpb2* for 1 min 30 s); with an extension at 72 °C for 2 min; and a final extension at 72 °C for 10 min.

PCR products were purified and sequenced using the same primers (Table 1) by SolGent Co. (South Korea) and Sangon Biotech (Shanghai, China).

### Phylogenetic analyses

Raw sequence reads were verified in DNA Baser Assembler and ambiguous bases at the 5' and 3' ends were manually trimmed. Consensus sequences were assembled in SeqMan (DNAStar, Madison, Wisconsin, USA). All sequences were deposited in the NCBI GenBank database, with accession numbers provided for each strain (Table 2). Newly-generated sequences were subjected to BLASTn searches and corresponding reference sequences were retrieved from GenBank.

**Table 2.** List of isolates and strains included in the DNA analyses with their GenBank accession numbers. Strains generated in this study are in bold and ex-type/reference isolates and strains are marked with ^T^.

**Families**	**Species**	**Strain/Isolate number**	**28S**	**ITS**	**18S**	***tef1-α***	***rpb2***
Anastomitrabeculiaceae	***Anastomitrabeculia chiangraiensis***	**MFLU 26-0002 ^T^**	**PX956940**	**PX940740**	**–**	**–**	**PZ097584**
	***Anastomitrabeculia chiangraiensis***	**MFLU 26-0003**	**PX956941**	**PX940741**	**–**	**–**	**–**
	*Anastomitrabeculia didymospora*	MFLUCC 16-0412 **^T^**	MW412978	**–**	MW412977	MW411338	**–**
	*Anastomitrabeculia didymospora*	MFLUCC 16-0417	MW413899	**–**	**–**	MW411339	**–**
	*Anastomitrabeculia didymospora*	MFLUCC 11-0197	ON077068	ON077079	**–**	ON075062	**–**
	*Anastomitrabeculia didymospora*	MFLUCC 11-0200	ON077069	ON077080	ON077074	ON075063	ON075067
	*Anastomitrabeculia xishuangbannaensis*	KUMCC 21-0585 **^T^**	OQ170834	OQ158914	OQ168194	OR613420	**–**
	*Anastomitrabeculia xishuangbannaensis*	KUMCC 21-0586	OQ170835	OQ158915	OQ168195	**–**	**–**
Cyclothyriellaceae	*Cyclothyriella rubronotata*	CBS 141486 **^T^**	KX650544	NR_147651	NG_061252	KX650519	KX650574
	*Massariosphaeria clematidis*	MFLU 16-0174 **^T^**	MT214544	MT310591	MT226663	–	–
Corynesporascaceae	*Corynespora cassiicola*	CBS 100822	GU301808	–	GU296144	GU349052	GU371742
	*Corynespora torulosa*	CPC 15989 **^T^**	KF777207	NR_145181	–	–	–
Longiostiolaceae	*Crassiperidium octosporum*	MAFF 242971 **^T^**	LC373108	LC373096	LC373084	LC373120	LC373132
	*Crassiperidium octosporum*	MAFF 246401	LC373111	LC373099	LC373087	LC373123	LC373135
	*Crassiperidium octosporum*	KT 3008	LC373110	LC373098	LC373086	LC373122	LC373134
	*Crassiperidium octosporum*	KT 2894	LC373109	LC373097	LC373085	LC373121	LC373133
	*Crassiperidium quadrisporum*	KT 2798-2 **^T^**	LC373119	LC373107	LC373095	LC373131	LC373143
	*Crassiperidium quadrisporum*	KT 2798-1	LC373118	LC373106	LC373094	LC373130	LC373142
	*Longiostiolum_tectonae*	MFLUCC 12 0562 **^T^**	KU764700	KU712447	KU712459	–	–
	*Shearia formosa*	HKAS107010	OQ703052	OR053807	OQ703046	OQ708651	OQ766961
	*Shearia formosa*	HKAS107033	OQ703055	OR053809	OQ703049	OQ708654	OQ766962
	*Shearia formosa*	MFLU 19-2114	OQ703054	OR053805	OQ703048	OQ708652	OQ708657
	*Shearia formosa*	HKAS107011	OQ703053	OR053808	OQ703047	OQ708656	OQ766960
	*Shearia formosa*	MFLU 19-2115	OQ703057	OR053806	OQ703051	OQ708653	OQ766959
	*Shearia formosa*	HKAS107034	OQ703056	OR053810	OQ703050	OQ708655	OQ766963
	*Shearia formosa*	MFLUCC 20-0018	MT159621	MT159627	MT159633	MT159604	MT159610
	*Shearia formosa*	MFLUCC 20-0019	MT159620	MT159626	MT159632	MT159603	MT159609
	*Shearia formosa*	GMBCC 1172	OM855601	OM855592	OM855615	–	OM857557
	*Shearia formosa*	MFLUCC 20-0017	MT159619	MT159625	MT159631	MT159602	MT159608
Roussoellaceae	*Roussoella angusta*	MFLUCC 15-0186 **^T^**	KT281979	–	–	–	–
	*Roussoella aquatica*	MFLUCC 18-1040 **^T^**	MN913707	MT627703	MT864297	–	–
	*Roussoella arundinacea*	CBS 146088 **^T^**	MT223928	MT223838	–	–	MT223699
	*Roussoella aseptata*	UESTCC 24.0151	PQ067710	PQ067879	PQ066552	PQ278560	–
	*Roussoella aseptata*	CGMCC 3.25604 **^T^**	PQ067709	PQ067878	PQ066551	–	–
	*Roussoella bambusarum*	GMB0316 **^T^**	ON479892	ON479891	–	–	–
	*Roussoella bambusarum*	GMB0390	ON505051	ON505055	–	–	–
	*Roussoella bambusicola*	KUMCC:21-0055a	PX612207	PX612195	PX612203	–	PX556783
	*Roussoella bambusicola*	KUMCC 21-0055b	PX612208	PX612196	PX612204	–	PX556784
	***Roussoella chiangraiensis***	**MFLU 26-0004 ^T^**	**PX956942**	**PX940742**	**PX956882**	**–**	**PZ097585**
	*Roussoella chiangraina*	MFLUCC 10-0556 **^T^**	KJ474840	KJ474828	–	KJ474849	KJ474857
	*Roussoella chinensis*	KUNCC 22-12534 **^T^**	OP555449	OP555451	OP555446	–	–
	*Roussoella doimaesalongensis*	MFLUCC 14-0584 **^T^**	KY000659	KY026584	–	KY651249	KY678394
	*Roussoella dujuanhuensis*	HKAS 131782 **^T^**	PP189909	PQ340471	PQ435266	PQ456961	PQ348603
	*Roussoella fusispora*	UESTCC 23.0135 **^T^**	OR142438	OR141720	–	OR161835	OR161837
	*Roussoella guizhouensis*	GUCC 24-0199 **^T^**	PQ475845	PQ404882	–	PQ438563	PQ399769
	*Roussoella guizhouensis*	GUCC 24-0200	PQ475847	PQ404883	–	PQ438564	PQ399770
Roussoellaceae	*Roussoella guizhouensis*	GUCC 24-0201	PQ475846	PQ404884	–	PQ438565	PQ399771
	*Roussoella guttulata*	MFLUCC 20-0102 **^T^**	NG_075383	NR_172428	–	MW022188	MW022187
	*Roussoella hydei*	GMB-W1191 **^T^**	PV022509	PV022386	–	PV452824	–
	*Roussoella hydei*	GMB-W1192	PV022510	PV022387	–	PV452825	–
	*Roussoella hysterioides*	CBS 546.94	KF443381	KF443405	AY642528	KF443399	KF443392
	*Roussoella intermedia*	CBS 170.96	KF443382	KF443407	KF443390	KF443398	KF443394
	*Roussoella japanensis*	MAFF 239636 **^T^**	AB524621	KJ474829	AB524480	AB539114	AB539101
	*Roussoella japanensis*	GMBCC 1067	OM884018	OM891802	OM891823	ON098344	ON098381
	*Roussoella japanensis*	GMBCC 1117	OM884023	OM891811	OM891831	ON098345	ON098382
	*Roussoella kunmingensis*	KUMCC 18-0128 **^T^**	MH453487	MH453491	–	MH453480	MH453484
	*Roussoella kunmingensis*	GMBCC 1055	OM884013	OM891797	–	ON098353	ON098385
	*Roussoella magnata*	MFLUCC 15-0185 **^T^**	KT281980	–	–	–	–
	*Roussoella margidorensis*	MUT 5329 **^T^**	MN556322	KU314944	MN556309	MN605897	MN605917
	*Roussoella marietteae*	BRIP 70563 **^T^**	OM333605	OM417291	–	ON624200	ON624228
	*Roussoella marietteae*	BRIP 76677a	PQ793834	PQ793835	–	–	–
	*Roussoella mediterranea*	MUT 5369 **^T^**	MN556324	KU314947	KU314948	MN605899	MN605919
	*Roussoella mexicana*	CPC 25355 **^T^**	KT950862	KT950848	–	–	–
	*Roussoella multiloculata*	GMBCC 1056 **^T^**	OM884015	OM891799	OM891821	ON098343	ON098369
	*Roussoella multiloculata*	GMBCC 1065	OM884016	OM891800	ON124715	ON098338	ON098364
	*Roussoella neoaquatica*	HKAS 126513 **^T^**	OR762066	OR762065	–	OR763024	–
	*Roussoella neopustulans*	MFLUCC 11-0609 **^T^**	KJ474841	KJ474833	–	KJ474850	–
	*Roussoella neopustulans*	MFLUCC 12-0003	KU863119	KU940130	–	–	–
	*Roussoella nitidula*	MFLUCC 11-0182 **^T^**	KJ474843	KJ474835	–	KJ474852	KJ474859
	*Roussoella nitidula*	MFLUCC 11-0634	KJ474842	KJ474834	–	KJ474851	KJ474858
	*Roussoella nitidula*	GMBCC 1097	OM884020	OM891805	OM891826	ON098351	ON098384
	*Roussoella padinae*	MUT 5503 **^T^**	MN556327	KU158170	MN556312	MN605902	MN605922
	*Roussoella panzhouensis*	HKAS 136892 **^T^**	–	PQ325282	–	PQ761132	–
	*Roussoella papillata*	GMBCC 1121 **^T^**	OM755608	OM891814	–	ON098346	ON098378
	*Roussoella papillata*	IFRDCC3103	ON228184	ON228188	ON228186	ON244452	ON244450
	*Roussoella pseudohysterioides*	MFLUCC 13-0852 **^T^**	KU863120	KU940131	KU872123	KU940198	–
	*Roussoella pseudohysterioides*	KUMCC 18-0111	MH453486	MH453490	–	MH453479	MH453483
	*Roussoella pustulans*	MAFF 239637 **^T^**	AB524623	KJ474830	AB524482	AB539116	AB539103
	*Roussoella saprophytica*	CGMCC 3.25601 **^T^**	PQ067704	PQ067874	PQ066547	PQ278556	PQ186974
	*Roussoella saprophytica*	UESTCC 24.0150	PQ067706	–	PQ066548	–	–
	*Roussoella scabrispora*	MFLUCC 11-0624 **^T^**	KJ474844	KJ474836	–	KJ474853	KJ474860
	*Roussoella scabrispora*	MFLUCC 14-0582	KY000660	KY026583	–	–	–
	*Roussoella siamensis*	MFLUCC 11-0149 **^T^**	KJ474845	KJ474837	–	KJ474854	KJ474861
	*Roussoella sichuanensis*	UESTCC 23.0136 **^T^**	OR142439	OR141721	–	OR161836	OR161838
	*Roussoella sinensis*	GMBCC 1119 **^T^**	OM884024	OM891813	OM891833	ON098357	ON098379
	*Roussoella sinensis*	IFRDCC 3101	ON228183	ON228187	ON228185	ON244453	ON244451
	*Roussoella striata*	CGMCC 3.25603 **^T^**	PQ067708	PQ067877	PQ066550	PQ278559	PQ186975
	*Roussoella terrestris*	MFLUCC 24-0490 **^T^**	PQ577819	PP993960	–	PV624715	PV624713
	*Roussoella terrestris*	MFLUCC 24-0491	PQ577821	PQ577820	–	PV624714	–
	*Roussoella terricola*	CGMCC 3.25602 **^T^**	PQ067707	PQ067876	PQ066549	–	–
	*Roussoella thailandica*	MFLUCC 11-0621 **^T^**	KJ474846	KJ474838	–	–	–
	*Roussoella tuberculata*	MFLUCC 13-0854 **^T^**	KU863121	KU940132	KU872124	KU940199	–
	*Roussoella tuberculata*	GMBCC 1123	OM755613	OM891815	OM891834	ON098352	ON098380
	*Roussoella uniloculata*	GMBCC 1110 **^T^**	OM801286	OM891809	OM891829	ON098360	ON098374
	*Roussoella uniloculata*	DDQ01005-2	OM884026	OM891817	–	ON098361	ON098375
	*Roussoella verrucispora*	CBS 125434 **^T^**	AB524622	KJ474832	AB524481	AB539115	AB539102
Roussoellaceae	*Roussoella yunnanensis*	KUMCC 18-0115 **^T^**	MH453488	MH453492	–	MH453481	–
Tubeufiaceae (Outgroups)	*Berkleasmium longisporum*	MFLUCC 17-1999 **^T^**	MH558825	MH558698	–	MH550889	MH551012
	*Berkleasmium longisporum*	MFLUCC 17-2002	MH558826	MH558699	–	MH550890	MH551013
	*Tubeufia hainanensis*	GZCC 22-2015 **^T^**	OR030835	OR030842	–	OR046679	OR046685
	*Tubeufia hainanensis*	GZCC 23-0589	OR066421	OR066414	–	OR058860	OR058853

–: Data not available.

Single-gene alignments were generated in MAFFT v.7 (Katoh et al. 2019) using default settings and trimmed with trimAl (Capella-Gutiérrez et al. 2009). The trimmed alignments were concatenated in SequenceMatrix (Vaidya et al. 2011). Maximum Likelihood analyses were performed on the IQ-TREE webserver with default parameters and 1000 ultrafast bootstrap replicates (Nguyen et al. 2015). Substitution models for each locus were selected independently using jModelTest v.2.1.6 under the Akaike Information Criterion (AIC).

Bayesian Inference was carried out in MrBayes v.3.2.7a (Huelsenbeck et al. 2001; Ronquist and Huelsenbeck 2003). MCMC analyses used four parallel chains, sampled every 100 generations. A 20% burn-in was applied and the remaining trees were used to calculate posterior probabilities. Phylogenetic trees were visualised in FigTree v.1.4.4 (Rambaut and Drummond 2014).

### Genetic distances

Interspecies genetic distances were estimated in MEGA-X using the Kimura 2-parameter + gamma model with pairwise deletion to support the taxonomic placement of newly described taxa (Tamura et al. 2013).

## Results

**Figure 1.**
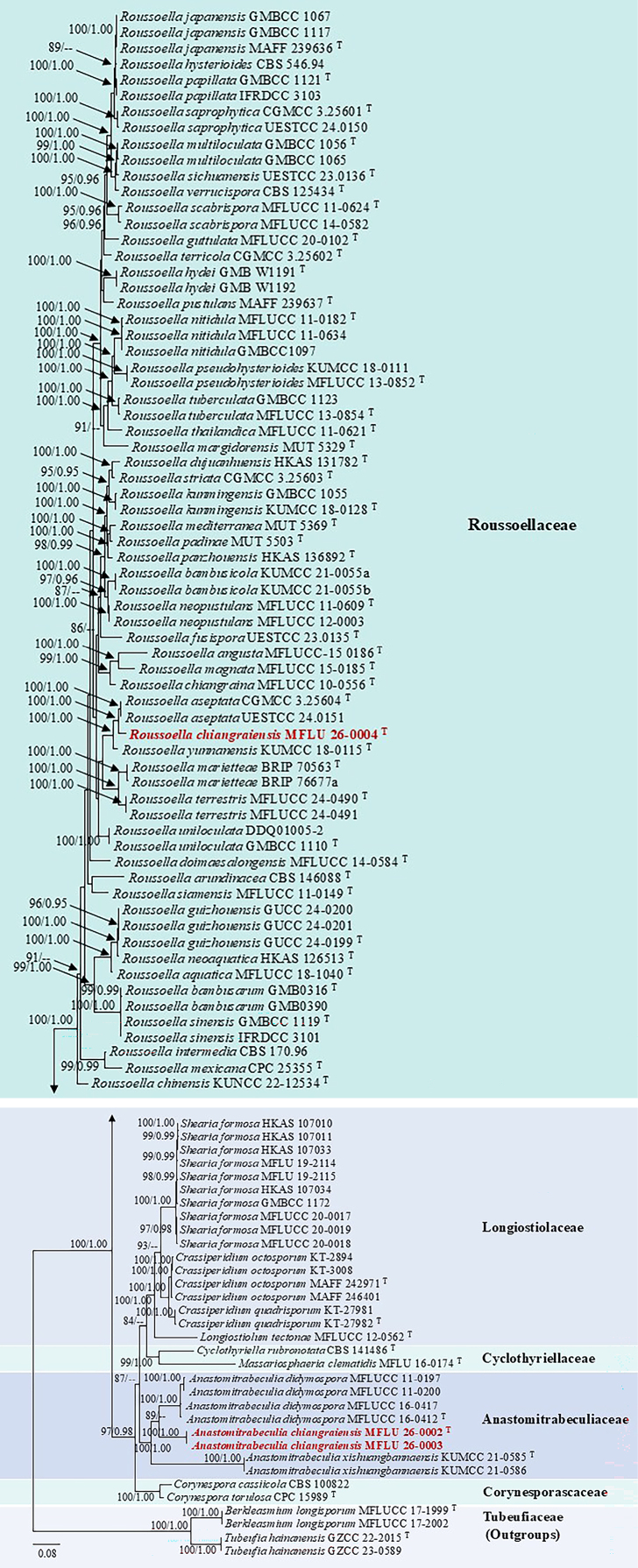
Maximum Likelihood (ML) phylogeny inferred from a combined alignment of 28S, ITS, 18S, *tef1-α* and *rpb2* sequences for representative taxa belonging to different families in Pleosporales. Branch labels show ML bootstrap values ≥ 80% and Bayesian posterior probabilities ≥ 0.95, presented as ML/PP. Double hyphens (--) denote nodes with support values below these thresholds. The tree is rooted with Tubeufia hainanensis (GZCC 22-2015 and GZCC 23-0589) and Berkleasmium longisporum (MFLUCC 17-1999 and MFLUCC 17-2002). Type, ex-type and reference isolates and strains are indicated with

Three strains representing two distinct novel taxa, one in *Anastomitrabeculia* and the other in *Roussoella (Pleosporales)*, were isolated. Each species was documented as a saprobe in its sexual morph and taxonomic descriptions are arranged alphabetically. The placements of the new species are supported by morphological characteristics and phylogenetic analyses. The phylogram obtained from the IQ-TREE analysis was used as the topological backbone, yielding an optimal log-likelihood value of -36250.874 (Fig. 1). The total tree length was 3.688. The final matrix comprised 4305 aligned characters (28S: 1–847 bp; ITS: 848–1363 bp; 18S: 1364–2347 bp; *tef1-α*: 2348–3269 bp; and *rpb2*: 3270–4305 bp). Among the 4305 characters, 1888 were distinct patterns, 1373 were parsimony-informative, 199 were singleton sites and 2733 were constant sites.

### Taxonomy

**Class *Dothideomycetes* O.E. Erikss & Winka**

**Subclass *Pleosporomycetidae* C.L. Schoch, Spatafora, Crous & Shoemaker**

***Pleosporales* Luttr. ex M.E. Barr**

***Anastomitrabeculiaceae* Bhunjun, Phukhams. & K.D. Hyde**

***Anastomitrabeculia* Bhunjun, Phukhams. & K.D. Hyde**

#### Anastomitrabeculia chiangraiensis

Taxon classificationFungiPleosporalesAnastomitrabeculiaceae

Gomdola, Z.L. Tun & Yong Wang bis sp. nov.

56404F2B-4AA1-58DE-B06E-D2FFE93F9F6E

Fig. 2

##### Holotype.

MFLU 26-0002.

##### Etymology.

Referring to Chiang Rai Province, where the species was collected.

##### Diagnosis.

Ascospores without longitudinal striations.

##### Description.

Saprobic on stems of an unidentified host. **Sexual morph: *Ascomata*** 350–450 μm diam. (x̄ = 405 μm, n = 5), 375–500 μm high (x̄ = 445 μm, n = 5), semi-immersed or immersed, solitary, aggregated or scattered, globose to subglobose, black, ostiolate. ***Ostiole*** 50–75 μm diam. (x̄ = 61.9 μm, n = 5), 45–80 μm high (x̄ = 72 μm, n = 5), central. ***Peridium*** 5.5–10 μm thick (x̄ = 7.4 μm, n = 5), composed of 4–6 layers of pale brown to brown ***textura angularis*** cells, with hyaline inner layers. ***Hamathecium*** consisting of dense, filiform, filamentous, trabeculate pseudoparaphyses. ***Pseudoparaphyses*** 0.5–2 μm wide (x̄ = 1.6 μm, n = 30), branched or unbranched, septate, hyaline. ***Asci*** (89–)128–197 × 15–21 μm (x̄ = 173 × 18.6 μm, n = 20), bitunicate, fissitunicate, 8-spored, cylindrical to clavate, straight or slightly curved, broadly rounded at the apex with an ocular chamber and mostly with a short pedicel [8–15(–19) μm long (x̄ = 11.1 μm, n = 20)]. ***Ascospores*** 25–30 × 7–8.5 µm (x̄ = 28.1 × 7.8 µm; n = 20), overlapping uni- to biseriate, hyaline, broadly fusiform or subcylindrical, broader at the center and tapering towards the ends, mostly straight, smooth-walled, 1-median-septate, constricted at the septum, containing two large guttules in each cell and surrounded by a mucilaginous sheath of nearly equal thickness [0.7–1.8 μm long (x̄ = 1.2 μm, n = 20)]. **Asexual morph**: Not observed.

**Figure 2.**
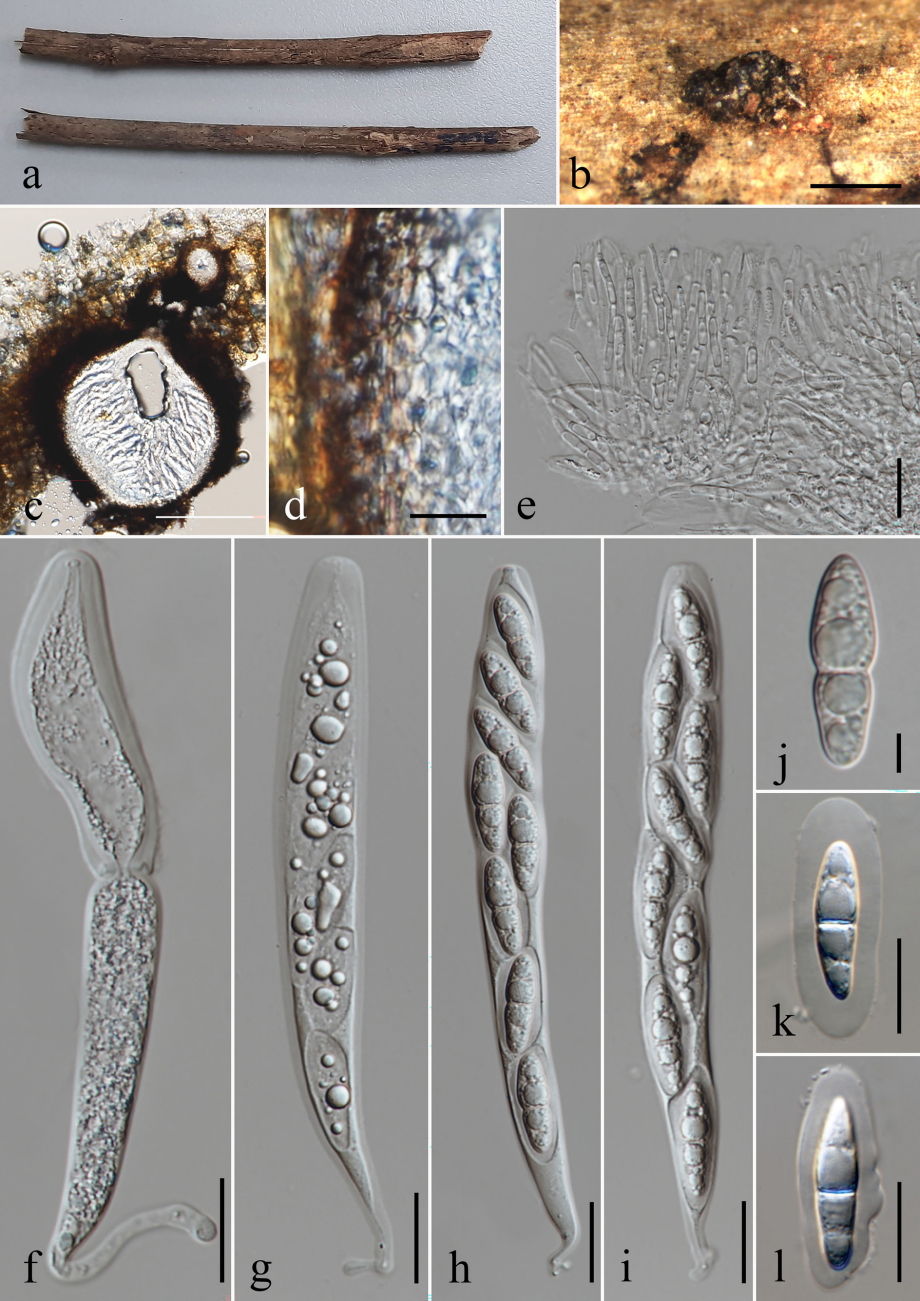
*Anastomitrabeculia chiangraiensis* (MFLU 26-0002, new species). **a**. Substrates; **b**. An ascoma on the substrate; **c**. Section through an ascoma; **d**. Peridium; **e**. Pseudoparaphyses; **f, g**. Immature asci; **h, i**. Mature asci; **j**. An ascospore; **k, l**. Ascospores surrounded by a mucilaginous sheath. Scale bars: 1 mm (**b**); 200 µm (**c**); 5 µm (**d, j–l**); 20 µm (**e–i**).

##### Material examined.

Thailand. • Chiang Rai Province: Mae Fah Luang University premises, on dead stems of an unidentified host, 15 June 2024, Zaw Lin Tun, P21 (MFLU 26-0002, holotype). Same details, P21-B (MFLU 26-0003).

##### GenBank numbers.

MFLU 26-0002: ITS = PX940740, 28S = PX956940, *rpb2* = PZ097584; MFLU 26-0003: ITS = PX940741, 28S = PX956941.

##### Notes.

Our strains (MFLU 26-0002 and MFLU 26-0003) conform to the morphological species concept of *Anastomitrabeculia*, which is characterised by bitunicate, fissitunicate and cylindrical to clavate asci with an ocular chamber, and hyaline, 1-septate, broadly fusiform ascospores that taper towards the ends and are surrounded by a mucilaginous sheath (Bhunjun et al. 2021).

Our strains clustered together with maximum support (100% ML, 1.00 PP) and formed a sister lineage to the clade comprising isolates of *A. didymospora* (MFLUCC 16-0412; ex-type, MFLUCC 16-0417, MFLUCC 11-0197 and MFLUCC 11-0200), with 89% ML bootstrap support (Fig. 1). Excluding gaps, significant interspecies genetic distances were observed: 19.1% in ITS (92/482 bp) between our strains and *A. didymospora* (MFLUCC 11-0197 and MFLUCC 11-0200), 4.6% in 28S (38/827 bp) between our strains and the ex-type of *A. didymospora* (MFLUCC 16-0412) and 15.4% in *rpb2* between our strain (MFLU 26-0002) and *A. didymospora* (MFLUCC 11-0200). Morphologically, the most distinguishable feature separating our strains from *A. didymospora* is the presence of longitudinal striations in the ascospores, a character not observed in our strains (Bhunjun et al. 2021; Phookamsak et al. 2022).

Morphological data and multigene phylogenetic analyses sup­port the establishment MFLU 26-0002 and MFLU 26-0003 as a new species, *Anastomitrabeculia chiangraiensis*, saprobic on stems of an unidentified host in Thailand. This represents the third species in the genus.

### *Roussoellaceae* Jian K. Liu, Phook., D.Q. Dai & K.D. Hyde

***Roussoella* Sacc**.

#### Roussoella chiangraiensis

Taxon classificationFungiPleosporalesRoussoellaceae

Gomdola, Z.L. Tun & Yong Wang bis sp. nov.

2939B987-E3BB-571D-8DD6-E72F31689029

Fig. 3

##### Holotype.

MFLU 26-0004.

##### Etymology.

Referring to Chiang Rai Province, where the species was collected.

##### Description.

Saprobic on bamboo stems. **Sexual morph: *Ascomata*** 50–200 μm diam., superficial on the host substrate, aggregated, globose to subglobose, black. ***Asci*** 45–75 × 2.5–4 μm (x̄ = 60.1 × 3.6 μm, n = 20), bitunicate, fissitunicate, 6–8-spored, cylindrical to clavate, straight or slightly curved, rounded at the apex with an ocular chamber. ***Ascospores*** 6–10 × 2–3.5 µm (x̄ = 7.9 × 2.9 µm, n = 30), uniseriate, broadly fusiform to sub-ellipsoidal, tapering towards one or both ends, mostly straight, smooth- and thick-walled, hyaline when immature, becoming pale brown to brown at maturity, 1-median-septate, constricted at the septum, usually containing a large guttule in each cell, with longitudinal striations. **Asexual morph**: Not observed.

**Figure 3.**
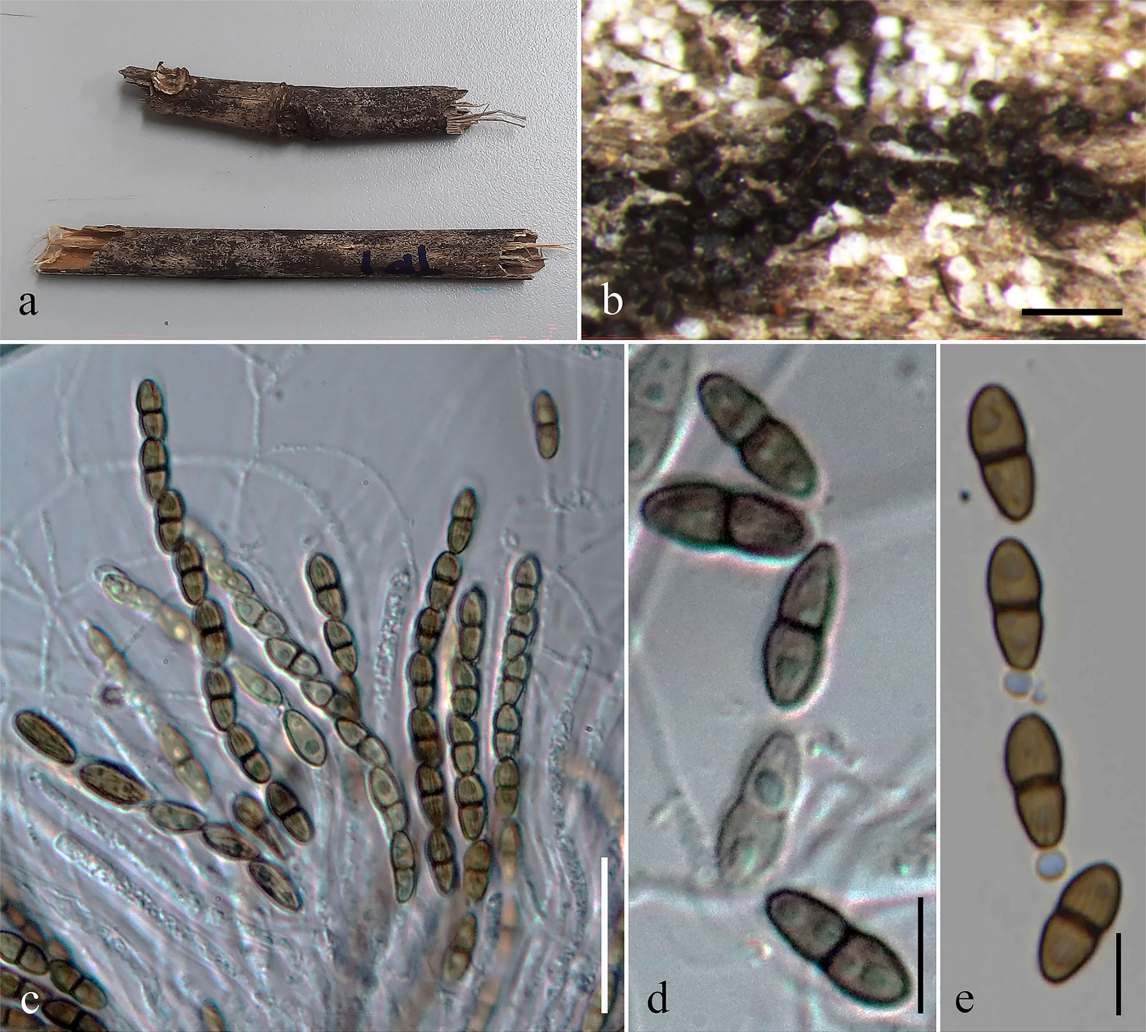
*Roussoella chiangraiensis* (MFLU 26-0004, new species). **a**. Substrates; **b**. Ascomata on the substrate; **c**. Asci; **d, e**. Ascospores. Scale bars: 250 µm (**b**); 20 µm (**c**); 5 µm (**d, e**).

##### Material examined.

Thailand. • Chiang Rai Province: Mae Fah Luang University premises, on dead stems of bamboo, 20 November 2025, Zaw Lin Tun, TP1 (MFLU 26-0004, holotype).

##### GenBank numbers.

ITS = PX940742, 28S = PX956942, 18S = PX956882, *rpb2* = PZ097585.

##### Notes.

Our strain (MFLU 26-0004) exhibits the diagnostic characters of *Roussoella*, including bitunicate asci, and brown, fusiform to ellipsoidal, septate ascospores (Liu et al. 2014). In the multilocus phylogeny, it forms a sister clade to *R. aseptata* (CGMCC 3.25604; ex-type and UESTCC 24.0151), with maximum support (100% ML, 1.00 PP) (Fig. 1). No nucleotide differences were detected in the 18S region between our strain and *R. aseptata* (CGMCC 3.25604; 622 bp and UESTCC 24.0151; 772 bp). However, excluding gaps, interspecies divergences of 3.9% in ITS (18/462 bp) and 0.24% in 28S (2/845 bp) were recovered.

Morphological comparison with *R. aseptata* was not possible, as the latter is known only from its asexual morph, whereas our strain was obtained in the sexual morph (Yu et al. 2024). Based primarily on phylogenetic analyses and the ITS divergence, we describe MFLU 26-0004 as a new species, *Roussoella chiangraiensis*, saprobic on bamboo stems in Thailand.

It is noteworthy that our strain lacks the raised, semi-immersed to immersed ascostromata typical of many *Roussoella* species (Liu et al. 2014). Instead, it produces superficial, carbonaceous ascomata that could not be hand-sectioned and appear among other free fungal spores on the substrate.

## Discussion

Our study introduces two novel Pleosporalean species from northern Thailand: *Anastomitrabeculia chiangraiensis* and *Roussoella chiangraiensis*. Both species were recovered from dead plant substrates and exhibit diagnostic morphological features consistent with their respective genera. Multigene phylogenetic analyses further confirm their distinctiveness, supporting their recognition as new species.

As listed in Species Fungorum (2026), *Anastomitrabeculia (Anastomitrabeculiaceae)* remains poorly represented, with only two epithets to date. On the other hand, *Roussoella* comprises 70 epithets, reflecting its status as a relatively species-rich genus within *Roussoellaceae*. The striking disparity in species richness between these genera highlights several ecological and taxonomic considerations.

The low diversity recorded for *Anastomitrabeculia* may partly reflect insufficient sampling rather than true biological scarcity. The genus is known to produce immersed ascomata (Bhunjun et al. 2021; Phookamsak et al. 2022) and conidiomata (Ren et al. 2024) that are often inconspicuous on the host surface, making them easily overlooked during field surveys. Such cryptic fruiting bodies can lead to underestimation of diversity, particularly in environments where woody substrates harbour many co-occurring microfungi. Additionally, *Anastomitrabeculia* appears to be geographically restricted or at least rarely reported, possibly due to limited targeted investigations within *Anastomitrabeculiaceae*. The discovery of *A. chiangraiensis* from dead wood in Thailand suggests that additional species may remain undiscovered in subtropical and tropical forests, especially where dead woody substrates are abundant.

In contrast, the comparatively high diversity of *Roussoella* may be attributed to several factors. First, *Roussoella* species frequently colonise bamboo, a widespread and taxonomically diverse host group in Asia (Dai et al. 2017; Sun et al. 2025). The structural complexity and fast turnover of bamboo culms create numerous microhabitats suitable for saprobic colonisation. Second, *Roussoella* species typically form ascostromata that are mostly immersed to erumpent through the host surface, increasing their detectability during routine surveys (Sun et al. 2025). Third, the genus has been the focus of extensive taxonomic revision in recent years, with integrative morphological and molecular studies contributing to frequent species descriptions (Dai et al. 2017, 2022; Jiang et al. 2019; Sun et al. 2025). The addition of *R. chiangraiensis* further highlights the genus’s strong association with bamboo and reinforces the importance of bamboo-rich ecosystems as reservoirs of fungal biodiversity.

The two new species introduced here exemplify the hidden diversity of saprobic *Pleosporales* in Southeast Asia. Their discovery underscores the value of comprehensive sampling coupled with multigene phylogenetic analysis for resolving species boundaries in morphologically conserved genera. Moreover, these findings contribute to a growing understanding of host- and substrate-associated diversity patterns in *Pleosporales*, particularly within *Anastomitrabeculiaceae* and *Roussoellaceae*. Continued research on decaying plant substrates, including less conspicuous microhabitats, is likely to yield additional taxa and refine our understanding of fungal distribution. The present study highlights Thailand, particularly the north, as a promising region for further fungal discovery, especially in habitats dominated by bamboo and mixed woody vegetation. Ultimately, documenting such diversity is essential for establishing a more comprehensive and stable taxonomy of *Pleosporales*.

## Supplementary Material

XML Treatment for Anastomitrabeculia chiangraiensis

XML Treatment for Roussoella chiangraiensis

## Additional information

### Artificial Intelligence (AI) use

The authors accept full responsibility for the content of the manuscript, including the disclosure of any use of AI.

No AI tools were used in the preparation of this manuscript.

### Author ORCIDs

Z.L. Tun https://orcid.org/0009-0001-3108-4425

Y. Wang https://orcid.org/0000-0003-3831-2117

D. Gomdola https://orcid.org/0000-0002-0817-1555

L.-F. Yu https://orcid.org/0009-0004-5157-5143

### Data availability

All data generated or analysed during this study are included in this published article.

## References

[B1] Bhunjun CS, Phukhamsakda C, Jeewon R, Promputtha I, Hyde KD (2021) Integrating different lines of evidence to establish a novel ascomycete genus and family (*Anastomitrabeculia*, *Anastomitrabeculiaceae*) in *Pleosporales*. Journal of Fungi 7(2): е94. 10.3390/jof7020094 PMC7912389 33525387

[B2] Capella-Gutiérrez S, Silla-Martínez JM, Gabaldón T (2009) trimAl: A tool for automated alignment trimming in large-scale phylogenetic analyses. Bioinformatics 25(15): 1972–1973. 10.1093/bioinformatics/btp348 PMC2712344 19505945

[B3] Chaiwan N, Gomdola D, Wang S, Monkai J, Tibpromma S, Doilom M, Wanasinghe DN, Mortimer PE, Lumyong S, Hyde KD (2021) An online database providing updated information of microfungi in the Greater Mekong Subregion. Mycosphere 12(1): 1513–1526. 10.5943/mycosphere/12/1/19

[B4] Chethana KWT, Manawasinghe IS, Hurdeal VG, Bhunjun CS, Appadoo MA, Gentekaki E, Raspé O, Promputtha I, Hyde KD (2021) What are fungal species and how to delineate them? Fungal Diversity 109: 1–25. 10.1007/S13225-021-00483-9

[B5] Dai DQ, Phookamsak R, Wijayawardene NN, Li WJ, Bhat DJ, Xu JC, Taylor JE, Hyde KD, Chukeatirote E (2017) Bambusicolous fungi. Fungal Diversity 82: 1–105. 10.1007/s13225-016-0367-8

[B6] Dai DQ, Wijayawardene NN, Dayarathne MC, Kumla J, Han LS, Zhang GQ, Zhang X, Zhang TT, Chen HH (2022) Taxonomic and phylogenetic characterizations reveal four new species, two new asexual morph reports, and six new country records of bambusicolous *Roussoella* from China. Journal of Fungi 8: е532. 10.3390/jof8050532 PMC9145633 35628787

[B7] Doehlemann G, Ökmen B, Zhu W, Sharon A (2017) Plant pathogenic fungi. In: Heitman J, Howlett BJ, Crous PW, Stukenbrock EH, James TY, Gow NAR (Eds) The Kingdom Fungi. Chapter 34. 10.1128/9781555819583.ch34

[B8] Hongsanan S, Hyde KD, Phookamsak R, Wanasinghe DN, McKenzie EHC, Sarma VV, Boonmee S, Lücking R, Bhat DJ, Liu NG, Tennakoon DS, Pem D, Karunarathna A, Jiang SH, Jones EBG, Phillips AJL, Manawasinghe IS, Tibpromma S, Jayasiri SC, Sandamali DS, Jayawardena RS, Wijayawardene NN, Ekanayaka AH, Jeewon R, Lu YZ, Dissanayake AJ, Zeng XY, Luo ZL, Tian Q, Phukhamsakda C, Thambugala KM, Dai DQ, Chethana KWT, Samarakoon MC, Ertz D, Bao DF, Doilom M, Liu JK, Pérez-Ortega S, Suija A, Senwanna C, Wijesinghe SN, Konta S, Niranjan M, Zhang SN, Ariyawansa HA, Jiang HB, Zhang JF, Norphanphoun C, de Silva NI, Thiyagaraja V, Zhang H, Bezerra JDP, Miranda-González R, Aptroot A, Kashiwadani H, Harishchandra D, Sérusiaux E, Aluthmuhandiram JVS, Abeywickrama PD, Devadatha B, Wu HX, Moon KH, Gueidan C, Schumm F, Bundhun D, Mapook A, Monkai J, Chomnunti P, Suetrong S, Chaiwan N, Dayarathne MC, Yang J, Rathnayaka AR, Bhunjun CS, Xu JC, Zheng JS, Liu G, Feng Y, Xie N (2020) Refined families of *Dothideomycetes*: *Dothideomycetidae* and *Pleosporomycetidae*. Mycosphere 11(1): 1553–2107. 10.5943/mycosphere/11/1/13

[B9] Huelsenbeck JP, Ronquist F, Nielsen R, Bollback JP (2001) Bayesian inference of phylogeny and its impact on evolutionary biology. Science 294(5550): 2310–2314. 10.1126/science.1065889 11743192

[B10] Hyde KD, Jones EBG, Liu JK, Ariyawansa H, Boehm E, Boonmee S, Braun U, Chomnunti P, Crous PW, Dai DQ, Diederich P, Dissanayake A, Doilom M, Doveri F, Hongsanan S, Jayawardena R, Lawrey JD, Li YM, Liu YX, Lücking R, Monkai J, Muggia L, Nelsen MP, Pang KL, Phookamsak R, Senanayake IC, Shearer CA, Suetrong S, Tanaka K, Thambugala KM, Wijayawardene NN, Wikee S, Wu HX, Zhang Y, Aguirre-Hudson B, Alias SA, Aptroot A, Bahkali AH, Bezerra JL, Bhat DJ, Camporesi E, Chukeatirote E, Gueidan C, Hawksworth DL, Hirayama K, de Hoog S, Kang JC, Knudsen K, Li WJ, Li XH, Liu ZY, Mapook A, McKenzie EHC, Miller AN, Mortimer PE, Phillips AJL, Raja HA, Scheuer C, Schumm F, Taylor JE, Tian Q, Tibpromma S, Wanasinghe DN, Wang Y, Xu JC, Yacharoen S, Yan JY, Zhang M (2013) Families of *Dothideomycetes*. Fungal Diversity 63: 1–313. 10.1007/s13225-013-0263-4

[B11] Hyde KD, Norphanphoun C, Chen J, Dissanayake AJ, Doilom M, Hongsanan S, Jayawardena RS, Jeewon R, Perera RH, Thongbai B, Wanasinghe DN, Wisitrassameewong K, Tibpromma S, Stadler M (2018) Thailand’s amazing diversity: up to 96% of fungi in northern Thailand may be novel. Fungal Diversity 93: 215–239. 10.1007/s13225-018-0415-7

[B12] Hyde KD, Xu J, Rapior S, Jeewon R, Lumyong S, Niego AGT, Abeywickrama PD, Aluthmuhandiram JVS, Brahamanage RS, Brooks S, Chaiyasen A, Chethana KWT, Chomnunti P, Chepkirui C, Chuankid B, de Silva NI, Doilom M, Faulds C, Gentekaki E, Gopalan V, Kakumyan P, Harishchandra D, Hemachandran H, Hongsanan S, Karunarathna A, Karunarathna SC, Khan S, Kumla J, Jayawardena RS, Liu JK, Liu N, Luangharn T, Macabeo APG, Marasinghe DS, Meeks D, Mortimer PE, Mueller P, Nadir S, Nataraja KN, Nontachaiyapoom S, O’Brien M, Penkhrue W, Phukhamsakda C, Ramanan US, Rathnayaka AR, Sadaba RB, Sandargo B, Samarakoon BC, Tennakoon DS, Siva R, Sriprom W, Suryanarayanan TS, Sujarit K, Suwannarach N, Suwunwong T, Thongbai B, Thongklang N, Wei D, Wijesinghe SN, Winiski J, Yan J, Yasanthika E, Stadler M (2019) The amazing potential of fungi: 50 ways we can exploit fungi industrially. Fungal Diversity 97: 1–136. 10.1007/s13225-019-00430-9

[B13] Hyde KD, Noorabadi MT, Thiyagaraja V, He MQ, Johnston PR, Wijesinghe SN, Armand A, Biketova AY, Chethana KWT, Erdoğdu M, Ge ZW, Groenewald JZ, Hongsanan S et al. (2024) The 2024 Outline of *Fungi* and fungus-like taxa. Mycosphere 15: 5146–6239. 10.5943/mycosphere/15/1/25

[B14] Index Fungorum (2026) Index Fungorum. https://www.indexfungorum.org/Names/Names.asp [accessed 9 January 2026]

[B15] Jayasiri SC, Hyde KD, Ariyawansa HA, Bhat J, Buyck B, Cai L, Dai YC, Abd-Elsalam KA, Ertz D, Hidayat I, Jeewon R, Jones EBG, Bahkali AH, Karunarathna SC, Liu JK, Luangsa-ard JJ, Lumbsch HT, Maharachchikumbura SSN, McKenzie EHC, Moncalvo JM, Ghobad-Nejhad M, Nilsson H, Pang KL, Pereira OL, Phillips AJL, Raspé O, Rollins AW, Romero AI, Etayo J, Selçuk F, Stephenson SL, Suetrong S, Taylor JE, Tsui CKM, Vizzini A, Abdel-Wahab MA, Wen TC, Boonmee S, Dai DQ, Daranagama DA, Dissanayake AJ, Ekanayaka AH, Fryar SC, Hongsanan S, Jayawardena RS, Li WJ, Perera RH, Phookamsak R, de Silva NI, Thambugala KM, Tian Q, Wijayawardene NN, Zhao RL, Zhao Q, Kang JC, Promputtha I (2015) The Faces of Fungi database: Fungal names linked with morphology, phylogeny and human impacts. Fungal Diversity 74(1): 3–18. 10.1007/s13225-015-0351-8

[B16] Jiang HB, Hyde KD, Jayawardena RS, Doilom M, Xu J, Phookamsak R (2019) Taxonomic and phylogenetic characterizations reveal two new species and two new records of *Roussoella* (*Roussoellaceae*, *Pleosporales*) from Yunnan, China. Mycological Progress 18: 577–591. 10.1007/s11557-019-01471-9

[B17] Katoh K, Rozewicki J, Yamada KD (2019) MAFFT online service: Multiple sequence alignment, interactive sequence choice and visualization. Briefings in Bioinformatics 20(4): 1160–1166. 10.1093/bib/bbx108 PMC6781576 28968734

[B18] Liu JK, Phookamsak R, Dai DQ, Tanaka K, Jones EBG, Xu JC, Chukeatirote E, Hyde KD (2014) *Roussoellaceae*, a new pleosporalean family to accommodate the genera *Neoroussoella* gen. nov., *Roussoella* and *Roussoellopsis*. Phytotaxa 181(1): 1–33. 10.11646/phytotaxa.181.1.1

[B19] Liu Y, Whelen S, Hall BD (1999) Phylogenetic relationships amongst ascomycetes: Evidence from an RNA polymerase II subunit. Molecular Biology and Evolution 16(12): 1799–1808. 10.1093/oxfordjournals.molbev.a026092 10605121

[B20] Nguyen LT, Schmidt HA, von Haeseler A, Minh BQ (2015) IQ-TREE: A fast and effective stochastic algorithm for estimating maximum-likelihood phylogenies. Molecular Biology and Evolution 32(1): 268–274. 10.1093/molbev/msu300 PMC4271533 25371430

[B21] O’Hanlon R (2017) *Fungi* in the environment. In: Kavanagh K (Ed.) Fungi: Biology and Applications (3^rd^ ed.). Chapter 13. 10.1002/9781119374312.ch13

[B22] Phookamsak R, Jiang H, Suwannarach N, Lumyong S, Xu J, Xu S, Liao CF, Chomnunti P (2022) Bambusicolous fungi in *Pleosporales*: Introducing four novel taxa and a new habitat record for *Anastomitrabeculia didymospora*. Journal of Fungi 8: e630. 10.3390/jof8060630 PMC9225195 35736113

[B23] Rambaut A, Drummond A (2014) FigTree v1.3.1. Institute of Evolutionary Biology, University of Edinburgh.

[B24] Ren GC, Jayasiri SC, Tibpromma S, Farias ARG, Chethana KWT, Faraj KH, Wanasinghe DN, Xu JC, Hyde KD, Gui H (2024) Saprobic ascomycetes associated with woody litter from the Greater Mekong Subregion (Southwestern China and Northern Thailand). Mycosphere 15(1): 954–1082. 10.5943/mycosphere/15/1/8

[B25] Ronquist F, Huelsenbeck JP (2003) MrBayes 3: Bayesian phylogenetic inference under mixed models. Bioinformatics 19(12): 1572–1574. 10.1093/bioinformatics/btg180 12912839

[B26] Senanayake IC, Rathnayaka AR, Marasinghe DS, Calabon MS, Gentekaki E, Lee HB, Hurdeal VG, Pem D, Dissanayake LS, Wijesinghe SN, Bundhun D, Nguyen TT, Goonasekara ID, Abeywickrama PD, Bhunjun CS, Jayawardena RS, Wanasinghe DN, Jeewon R, Bhat DJ, Xiang MM (2020) Morphological approaches in studying fungi: Collection, examination, isolation, sporulation and preservation. Mycosphere 11(1): 2678–2754. 10.5943/mycosphere/11/1/20

[B27] Species Fungorum (2026) Species Fungorum. https://www.speciesfungorum.org/Names/Names.asp [accessed 9 January 2026]

[B28] Sun YR, Hyde KD, Liu NG, Jayawardena RS, Wijayawardene NN, Ma J, Zhang Q, Al-Otibi F, Wang Y (2025) Micro-fungi in southern China and northern Thailand: emphasis on medicinal plants. Fungal Diversity 131: 99–299. 10.1007/s13225-024-00549-4

[B29] Tamura K, Peterson D, Stecher G, Filipski A, Kumar S (2013) MEGA6: Molecular Evolutionary Genetics Analysis version 6.0. Molecular Biology and Evolution 30: 2725–2729. 10.1093/molbev/mst197 PMC3840312 24132122

[B30] Vaidya G, Lohman DJ, Meier R (2011) SequenceMatrix: Concatenation software for the fast assembly of multi-gene datasets with character set and codon information. Cladistics 27(2): 171–180. 10.1111/j.1096-0031.2010.00329.x 34875773

[B31] White TJ, Bruns T, Lee SJW, Taylor J (1990) Amplification and direct sequencing of fungal ribosomal RNA genes for phylogenetics. PCR Protocols 64: 315–322. 10.1016/B978-0-12-372180-8.50042-1

[B32] Yu XD, Zhang SN, Liang XD, Zhu JT, Hyde KD, Liu JK (2024) Bambusicolous fungi from Southwestern China. Mycosphere 15(1): 5038–5145. 10.5943/mycosphere/15/1/24

[B33] Zhang Y, Crous PW, Schoch CL, Hyde KD (2012) *Pleosporales*. Fungal Diversity 53(1): 1–221. 10.1007/s13225-011-0117-x PMC3477819 23097638

